# Experimental feasibility of personalized functional neuromuscular stimulation stepping patterns developed *in silico*

**DOI:** 10.3389/fbioe.2025.1609734

**Published:** 2025-07-30

**Authors:** Holly Warner, Musa L. Audu, Gabrielle C. Labrozzi, Nathaniel S. Makowski, Ronald J. Triolo

**Affiliations:** ^1^Department of Biomedical Engineering, Case Western Reserve University, Cleveland, OH, United States; ^2^Motion Study Laboratory, Louis Stokes Cleveland Veterans Affairs Medical Center, Cleveland, OH, United States; ^3^Department of Physical Medicine and Rehabilitation, MetroHealth System, Case Western Reserve University School of Medicine, Cleveland, OH, United States

**Keywords:** spinal cord injury, functional neuromuscular stimulation, neuroprosthesis, optimal control, musculoskeletal modeling, OpenSim

## Abstract

Functional neuromuscular stimulation is a technique for restoring mobility impaired by spinal cord injury, including stepping. Typically, functional neuromuscular stimulation patterns are determined by manually tuning stimulation timing and charge applied to peripheral nerves by modulating constant current pulse amplitude, width, or frequency. Manual tuning is time consuming and suboptimal; we propose an *in silico* alternative relying on optimal control for developing temporal patterns of stimulation that can be implemented in real-life functional neuromuscular stimulation systems. The functional neuromuscular stimulation system user model includes only those muscles available for activation with an existing functional neuromuscular stimulation system; optimal control goals and constraints emphasize simplicity to allow solutions to differ from neurotypical neuromuscular behavior. Reduction of stimulation levels and upper extremity effort during stepping are prioritized in the optimal control problem. A single study participant with incomplete spinal cord injury walked with both model-optimized and manually tuned functional neuromuscular stimulation patterns to determine the relative benefits of each. The optimized pattern reduced charge delivery by an average of 58% (35%–80% for eight of nine muscles) and improved the comfortability of left side muscle contractions. Relative to the manually tuned pattern, the model-optimized stimulation decreased upper extremity effort by 10.5% during left swing. Participant-informed modeling combined with optimal control could lead to efficient, personalized stimulation patterns.

## 1 Introduction

Spinal cord injury (SCI) is a life-changing event that interrupts many typical tasks such as trunk control, bladder and bowel function, walking, transfers, and weight shifting, among others. People living with lower-limb paralysis from thoracic level SCI have identified regaining the ability to walk as a priority among these tasks (Anderson, 2004; Ditunno et al., 2008; Lo et al., 2016; Ferber and Anderson, 2024). One method that can enable this ability is functional neuromuscular stimulation (FNS) (Kobetic and Marsolais, 1994; Knutson et al., 2006). FNS uses electrical currents to excite peripheral nerves that activate otherwise paralyzed muscles. FNS systems can be implemented with surgically implanted electrodes or electrodes adhered to the skin surface, depending on the needs and preferences of the user (Doucet et al., 2012).

An overarching control method must be selected to coordinate muscle activity for walking via FNS. Frequently, an open-loop, feedforward approach is taken, where the stimulation pattern is manually tuned on an individual basis (Kobetic and Marsolais, 1994). Manual tuning is practical for systems with only a few channels of FNS; however, as the number of neural targets increases, the complexity of coordination significantly expands. Coordinating muscles to achieve efficient gait is not simple, and typical patterns utilize maximal stimulation values to reduce the search space while guaranteeing joint stiffness (Kobetic and Marsolais, 1994). This maximal-stimulation approach can result in a muscle activation pattern that accelerates fatigue, rather than one that optimizes muscle-effort and generates stepping with metabolic efficiency. Maximal stimulation with open-loop feedforward control as in the manually tuned case also makes no allowance for modulating parameters beyond their saturation values in response to perturbations when compensating for system disturbance (Kuo, 2002). External perturbations such as a slip, trip, or incline can make an open-loop, feedforward stimulation pattern irrelevant to the circumstances and potentially destabilize the system (Kirsch et al., 2017). Internal perturbations like muscle fatigue can have a similar effect. Accordingly, it is desirable to decrease stimulation levels to allow for the addition of a feedback component to the control architecture to correct for changing situations of daily life (Blana et al., 2009). In summary, manual FNS tuning is laborious, fatigue-prone, and can limit the opportunity to implement feedback control.

To attain more energy efficient and personalized feedforward patterns for FNS-driven stepping, we adopted an *in silico* approach that uses optimal control (OC) techniques (Todorov and Li, 2003; Ackermann and van den Bogert, 2010) to determine minimal stimulation levels required to achieve the stepping task. *In silico* development of stimulation patterns could significantly simplify the personal customization process for each neuroprosthesis user, minimizing the time required and the fatigue associated with the trial-and-error method when manually tuning a pattern. By integrating model information into the solution process, OC can reduce computation time relative to alternative methods such as reinforcement learning. Furthermore, an OC approach could provide unique solutions that would not be identified in manual pattern tuning based on experience with neurotypical (NT) gait and thereby make better use of the patient-specific muscle set available via FNS. In addition to reducing stimulation levels, the pattern must not sacrifice other features of usability. Among these features we prioritized forward progression and reduced upper extremity effort (UEE) as optimization goals. This OC framework offers a systematic, *in silico* alternative to the manual tuning process.

Previous study has investigated OC for FNS. In (Santos et al., 2021) FNS for the prevention of post-stroke foot drop was considered in an OC context. Although the authors implemented a model with similar complexity to our work, they focused the use of FNS on controlling a single joint. To achieve gait for an individual with an SCI, multiple joints must be considered simultaneously. In feasibility studies of closing the loop around optimal control solutions, both the modeling and control were limited to a single joint and muscle. Kirsch et al. investigated a model-predictive control approach for knee extension driven by the quadriceps (Kirsch et al., 2017). Wang et al. studied an adaptive controller that minimized a cost function for the same joint and muscle combination (Wang et al., 2013). Multiple muscles were considered by Popovic et al. in a simulation-only study where a planar double-pendulum with four monoarticular muscles was modeled (Popovic et al., 1999). In this work we developed an OC algorithm within the context of a three-dimensional model containing mono- and bi-articular muscles, and evaluated its output experimentally in comparison to a manually tuned pattern in a long-term FNS system user with an incomplete SCI.

## 2 Methods

### 2.1 Model

Two OpenSim (Delp et al., 2007) musculoskeletal models were developed for this study. The first, a General Model (Model G), represented features of a generic individual with an SCI affecting the lower limbs who uses FNS. The second musculoskeletal model, a Study Participant-Informed Model (Model P), was developed by removing muscles from Model G. Model G ensured convergence of the optimal control problem, and Model P personalized the solution.

Model G was derived from (Zhao et al., 1998) (lower-limbs) and (Lambrecht et al., 2009) (upper body). The lower-limbs were modified from (Zhao et al., 1998) such that the resulting bipedal model was an open-chain linkage with six degrees of freedom (DOF) between the pelvis and ground. This was combined with the upper body model (Lambrecht et al., 2009) by use of a three DOF lumbosacral joint. The final model contained 29 DOF (three DOF per hip, one DOF per knee, two DOF per ankle, three DOF per shoulder, one DOF per elbow, and three DOF at the lumbosacral joint).

The boney structure of Model G and of Model P was scaled to the proportions of an NT volunteer (1.8415 m, 76.2 kg, 26 years); the intended volunteer with SCI was 1.778 m and 53.1 kg (42 years). Both individuals shared a similar athletic build. The NT volunteer had no neurological or musculoskeletal injuries or conditions that impair gait. We used the standard OpenSim scaling tool. For simplicity all contralateral segments were defined as symmetrical. From a static standing trial scaling factors are determined within OpenSim by measuring the distances between markers located on bony prominences. These scaling factors are then applied to the mass, inertial, and muscle geometry properties of the model by the OpenSim scaling tool. Additionally, the scaling tool determined the locations of the virtual markers on the model by a least-squares method (Delp et al., 2007).

Hill type muscle-tendon units actuated the model joints. The Hill muscle included force-length and force-velocity dependencies of muscle, passive muscle stiffness, tendon stiffness, and activation dynamics (Zajac, 1989). Muscle and tendon scaling parameters (maximum isometric force, optimal fiber length, and tendon slack length) were based on the literature (Delp et al., 1990; Lambrecht et al., 2009; Anderson, 2025). Due to the high degree of variation in muscle strength generated with FNS, we assumed NT values for the model maximum isometric force parameters. Scaling to match the strength of the study participant with SCI was completed during experimental testing as detailed in Section 2.7. For computational implementation we exchanged the source model’s Schutte1993 muscle formulation (Schutte et al., 1993), while maintaining the parameter values, for a more current OpenSim standard muscle model—the Millard2012EquilibriumMuscle (Millard et al., 2013). The Millard muscles were then converted by an OpenSim utility to the DeGrooteFregly2016Muscle model (De Groote et al., 2016), which allows for an implicit formulation of tendon dynamics used in solving the OC problem.

Each model included a reduced set of muscle elements compared to NT anatomy and gait (Table 1). Only muscles listed in Table 1 were represented (i.e., the passive properties of other muscles were assumed negligible because these muscles were not contracting). The Model G muscle set contained muscles commonly accessible by implanted stimulation systems (Hunt et al., 2017; Odle et al., 2019). The Model P muscle set was derived from Model G to contain muscles that had already been implanted with electrodes or could be recruited by surface stimulation with the study participant as indicated.

**TABLE 1:** Muscle groups for Model G and Model P and their associated stimulation method. Muscle locations are indicated as bilateral (B), left (L), and right (R). Stimulation methods are indicated as implanted (I) or surface (S).

Muscle group	Model		Stimulation method	
	G	P	Left	Right
Medial and Lateral Gastrocnemius and Soleus	B	-	-	-
Tibialis Anterior	B	B	I	I
Vastus Medialis, Intermedius, and Lateralis (Vasti)	B	B	I	I
Semimembranosus	B	-	-	-
Adductor Magnus (3 elements)	B	-	-	-
Gluteus Medius (3 elements)	B	-	-	-
Gluteus Maximus (3 elements)	B	-	-	-
Psoas and Iliacus	B	B	I	S
Erector Spinae	B	-	-	-
Rectus Abdominus	B	-	-	-
External Oblique	B	-	-	-
Quadratus Lumborum	B	-	-	-
Biceps Femoris Shorthead	-	R	-	S
Sartorius	-	R	-	S
Tensor Fasciae Latae	-	L	I	-

To represent upper body activity, the net activity of volitional muscle control, and participant interaction with a two-wheeled walker (wheels on front legs, tennis ball glides on back legs), the model included a set of force and torque actuators. Three DOF for each shoulder, elbow flexion, and lumbosacral roll and pitch were actuated in both models. “Reserve actuators” support convergence during solution of the OC problem, where dynamic inconsistencies might temporarily exist, and can provide input that represents the use of a walker aid. In Model G, reserve actuators were applied to the translational DOF for the pelvis. In transitioning from Model G to Model P, reserve actuators were introduced for the full six DOF of the pelvis, and an actuator was added for lumbosacral yaw.

Ground contact was represented with a smoothed form of the Hunt-Crossley contact model from OpenSim (Serrancoli et al., 2019; Dembia et al., 2020). This continuous and differentiable model is compatible with gradient-based solvers. To implement this contact model, a total of seven contact spheres were attached to each foot: two at the calcaneus, one on the lateral aspect of the arch, three attached distally across the metatarsals, and one at the center of the toes. A previous study indicated that the model is robust to the number, size, and placement of such contact elements (Falisse et al., 2019).

### 2.2 Optimal control

An OC problem is defined as finding the temporal control trajectory that minimizes a cost function and is subject to a set of constraints. The cost function and constraints are selected based on the problem of interest. For human motion studies, the cost function and constraints target physiological parameters, such as minimizing muscle effort or fatigue and limiting joint trajectories to feasible ranges.

OpenSim Moco was used to implement and solve a series of OC problems offline (Dembia et al., 2020). The Moco software package extends the OpenSim software by facilitating the combination of the OpenSim modeling tools with the direct collocation algorithm. Direct collocation discretizes both the trajectories of the system dynamics and the controls to formulate them as a set of algebraic equations that can be handled efficiently as a nonlinear program (Kelly, 2017). The OpenSim Moco package assembles OC problems with CasADi (Andersson et al., 2019) and solves the resulting nonlinear program with the gradient-based optimizer IPOPT (Wächter and Biegler, 2006). The OC problems were solved on a multi-core, mobile workstation computer operating an Intel^®^ Core™ i7-7700HQ CPU @ 2.80 GHz (Boost 3.80 GHz) processor with 4 cores (8 threads) and 16 GB RAM with parallel processing enabled.

### 2.3 NT reference data collection

Reference data collected from the NT individual described in Section 2.1 was used to inform tracking-related cost functions for the OC problem. Optical motion capture data (Vicon, Oxford, UK) sampled at 100 Hz and forceplate (AMTI, Watertown, MA) data sampled at 1000 Hz were recorded as an NT participant walked overground at a self-selected, slow pace. The study participant signed an informed consent form as approved by the Institutional Review Board of the Louis Stokes Cleveland VA Medical Center (Reference Number 1591730).

### 2.4 OC: Cost function subterms

Various subterms were combined to generate the cost function J . The cost function was evaluated across the period defined by the initial and final times $t_{i}$ and $t_{f}$ for the half or full gait cycle. We define each subterm of J here (Table 2) and describe their use in Section 2.6.

**TABLE 2:** Summary of cost function subterms.

Symbol	Purpose
$J_{mt}$	Marker tracking error
$J_{ct}$	Contact force tracking error
$J_{s}$	Control signal synergy within muscle groups
$J_{e}$	Minimize control effort
$J_{p}$	Penalize excess energy injection
$J_{ad}$	Minimize implicit auxiliary derivatives

To guide an OC problem toward a particular set of kinematics, a tracking term is commonly used. It compares kinematic model output to an experimentally measured kinematic output over time. For this case we used marker data from optical motion capture as the reference. The marker kinematic tracking subterm is represented by the cost function $J_{mt}$ :

$$J_{mt}=\int_{t_{i}}^{t_{f}}\sum_{k\in M}w_{k}{\left(x_{k,model}\left(t\right)-x_{k,meas}\left(t\right)\right)}^{2}dt$$ (1)

where M is the marker set, k is the index for individual markers in M , and $x_{k}$ is the three-dimensional marker position for either the model or the measured data. Weights w in Equation 1 were selected to emphasize bony prominences due to these locations’ higher marker placement accuracy: markers located at bony prominences were assigned weights a factor of ten higher than all other markers, which were equally weighted.

A second tracking subterm, contact tracking ( $J_{ct}$ ), was employed to ensure realistic ground contact forces. An equation comparing the contact forces output by the model and contact forces measured by a set of forceplates can be defined as

$$J_{ct}=\frac{1}{mg}\int_{t_{i}}^{t_{f}}\sum_{j\in G}{\left(F_{j,model}\left(t\right)-F_{j,meas}\left(t\right)\right)}^{2}dt$$ (2)

where G is the set of contact element groups (one per foot), j is the index for individual contact elements in G , $F_{j}$ is the vector sum of the three-dimensional forces for each contact element, m is the model mass, and g is the acceleration due to gravity in Equation 2.

Because a single channel activates all muscles or muscle elements in a group (groupings indicated by Table 1), we developed a cost function ( $J_{s}$ ) to impose synergy within a group:

$$J_{s}=\int_{t_{i}}^{t_{f}}\sum_{e\in S}{\left(e_{1}\left(t\right)-e_{2}\left(t\right)\right)}^{2}+{\left(e_{1}\left(t\right)-e_{3}\left(t\right)\right)}^{2}dt$$ (3)

where S is the set of muscle groups for which synergy applies, e is the muscle excitation, and subscripts 1, 2, and 3 represent individual muscle elements in a given muscle group. Where a pair of muscle elements define the group, only the first squared difference term in Equation 3 applies.

Control effort, which includes muscle excitation and reserve actuator control signals, can be minimized by the cost function subterm $J_{e}$ :

$$J_{e}=\frac{1}{d}\int_{t_{i}}^{t_{f}}\sum_{k\in M}w_{k}e_{k}^{2}\left(t\right)dt$$ (4)

where d is the distance travelled by the center of mass (Ackermann and van den Bogert, 2010). The weights w for the control signals in Equation 4 were selected such that reserve actuator activity was penalized. Because the reserve actuators represent use of the upper extremities via the walker, we wanted to encourage the solver to transfer as much of the effort required for stepping as possible to the lower limbs. All muscles were equally weighted.

The injection of extra energy, indicated by excessive trunk or pelvis motion, by the rectus abdominus ( RectAbd ) and external oblique ( ExtObl ) muscles can be corrected by the cost function subterm $J_{p}$ represented by Equation 5, which penalizes the muscle excitations for these muscles:

$$J_{p}=\int_{t_{i}}^{t_{f}}10\left(e_{RectAbd}^{2}\left(t\right)+e_{ExtObl}^{2}\left(t\right)\right)\;dt$$ (5)

Minimizing implicit auxiliary derivatives encourages convergence and was implemented by the cost function subterm $J_{ad}$ :

$$J_{ad}=\int_{t_{i}}^{t_{f}}\sum_{f\in T}{\left(\frac{dF_{f}}{dt}\right)}^{2}dt$$ (6)

where T is the set of tendons, f is the index for individual tendons in T , and $F_{f}$ is tendon force in Equation 6.

### 2.5 OC: Constraint terms

Multiple constraints were implemented for the solution of this OC problem. We define them generally here (Table 3) and specify their usage in Section 2.6.

**TABLE 3:** Summary of constraints.

Symbol	Purpose
$C_{sym,half}$	State and control symmetry for half stride
$C_{sym,full}$	State and control symmetry for full stride
$C_{speed\;bound}$	Bound joint speeds
$C_{avg\;speed}$	Average speed of center of mass
$C_{tf}$	Bound final time
$C_{intersect}$	Prevent legs intersecting
$C_{knees}$	Straighten knees to begin double stance
$C_{rest}$	Begin simulation from rest

Endpoint constraints enforced symmetry of the state and control values across either a half ( $C_{sym,half}$ ) or full stride ( $C_{sym,full}$ ). The lumbosacral and reserve actuators were exempt from $C_{sym,half}$ because they represent volitional effort and walker interaction, which are both controlled by the user.

To reduce the search space, we constrained the speed of each joint coordinate to remain within a selected percentage of the range for inverse kinematics (IK) computed from the NT reference data ( $C_{speed\;bound}$ ).

For OC problems where a tracking term was not considered in the cost function, constraints were used to promote forward motion. First, the average speed of the center of mass (CoM) was constrained ( $C_{avg\;speed}$ ), and second, the final time was assigned a set of bounds ( $C_{tf}$ ).

Additional constraints guided the solution toward a minimal set of desired features for predicting FNS-driven gait. These included preventing the legs from intersecting throughout the motion ( $C_{intersect}$ ), requiring straight knees (defined by a zero knee angle) when initiating double stance ( $C_{knees}$ ), and ensuring that the model starts with zero velocity for all initial velocities ( $C_{rest}$ ).

### 2.6 Optimized pattern development

**FIGURE 1:**
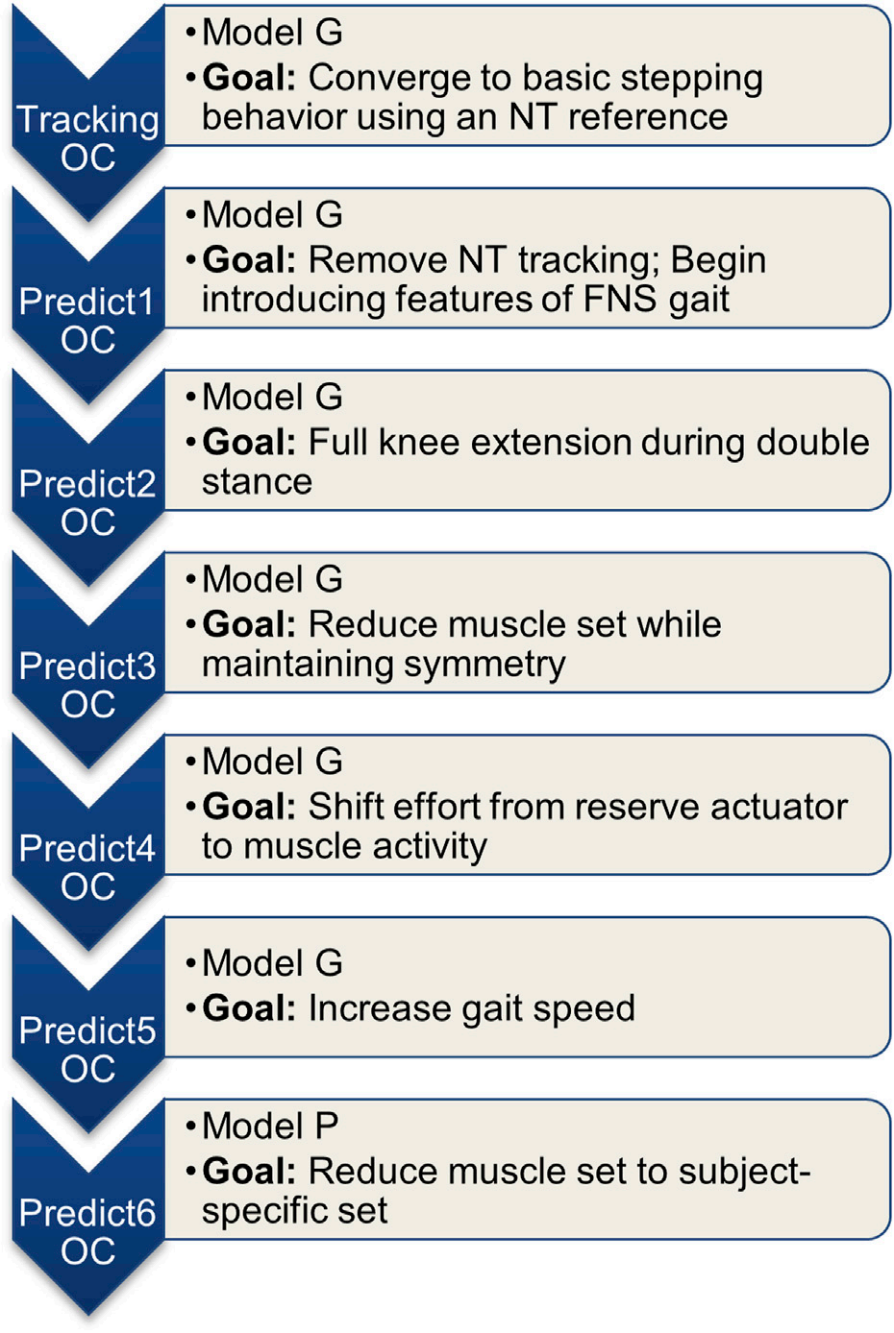
Summary of the series of OC problems solved. Each consecutive problem used the previous problem’s solution as the initial guess.

Multiple OC problems were solved in order to develop the final optimized pattern without an *a priori* initial guess. The optimized pattern development was completed in two stages. First, an OC tracking problem was solved to find a feasible initial guess for use in fully predictive (no tracking) OC problems. Second, a series of predictive OC problems were progressively refined toward the participant-specific conditions. This process is summarized in Figure 1.

The first stage of the OC problem solution solved an OC tracking problem. The OC tracking problem used Model G and included an NT reference for the cost subterms $J_{mt}$ and $J_{ct}$ . Energy minimization is a key feature of NT dynamic walking; therefore, we utilized NT tracking as a starting point to optimize FNS-driven gait (Cavagna and Kaneko, 1977). Note that when a tracking component is present, division of the effort cost subterm $J_{e}$ by the distance d is not necessary. The cost function for this case was composed as follows:

$$J_{NTtrack}=w_{mt}\;J_{mt}+w_{ct}\;J_{ct}+w_{s}\;J_{s}+d\;w_{e}\;J_{e}+w_{p}J_{p}+w_{ad}\;J_{ad}$$ (7)

where each w indicates the weight for a cost subterm. Constraints for the OC tracking problem included $C_{sym,half}$ and $C_{speed\;bound}=10\%$ of the reference NT values in Equation 7. When initializing the OC tracking problem, the initial guess was defined by the midpoint of variable bounds. During the first run, the problem was discretized across a grid of 10 mesh intervals. The solution for 10 mesh intervals was then used as the initial guess for a 50-mesh interval solution. All remaining optimizations were run at 50 mesh intervals.

In the second stage of the OC problem series, the first predictive OC problem used Model G and a cost similar to $J_{NTtrack}$ except that the tracking subterms were eliminated, as summarize by Equation 8.

$$J_{predict1}=w_{s}\;J_{s}+w_{e}\;J_{e}+w_{p}\;J_{p}+w_{ad}\;J_{ad}$$ (8)

Constraints were added to the set defined during the first stage: $C_{intersect}$ , $C_{rest}$ , $C_{avg\;speed}=0.4$ m/s, and $C_{tf}=\left[0.4,0.6\right]$ seconds, where values selected for $C_{avg\;speed}$ and $C_{tf}$ were based on the tracking solution used as the initial guess. In addition, $C_{speed\;bound}$ was expanded to 100% of the NT reference values to prevent incompatibility with other constraints while still providing bounds for the joint speed states.

Further development of the predictive optimization was then carried out in stage two, using each consecutive solution as the initial guess for the following OC problem. The subsequent OC problems were defined and solved in the following order:

Predict2) Added $C_{knees}$ to enforce full knee extension during double stance.
Predict3) Reduced the muscles to the symmetric set iliopsoas, vasti, and tibialis anterior. Due to the reduced muscle set, the desired average gait speed $C_{avg\;speed}$ was lowered to 0.1 m/s and consequently $C_{tf}$ was increased to [0.9, 1.1] seconds (which are typical for individuals with SCI using FNS). Also due to the updated muscle set, the actuator group was expanded to the full 6 DOF for the pelvis and 3 DOF for the lumbosacral joint.
Predict4) Increased the weight on the reserve actuators to reduce their activity.
Predict5) Raised prescribed average gait speed $C_{avg\;speed}$ to 0.2 m/s to increase gait speed observed in the previous solution.
Predict6) Revised model to Model P by adding the remaining muscles accessible by FNS for the participant. Due to the asymmetrical grouping of muscles, $C_{sym,half}$ was exchanged for $C_{sym,full}$ , and consequently, $C_{tf}$ was updated to [2.0, 2.2] seconds to accommodate a full two-step cycle.

### 2.7 Optimized pattern FNS sequence

Since the participant had volitional control over his stance limb muscles, we ignored the muscle excitation pattern solution during the stance phase. To obtain the swing phase portion of the optimized pattern, left-side muscle excitations were extracted from the first half of the optimization time period while right-side muscle excitations were extracted from the second half of the optimized time period.

For each muscle group (Table 1), the average across all muscle elements was computed to provide a single temporal sequence of muscle excitations for the group. The excitation sequences extracted for swing phase were then resampled using linear interpolation to 22 data points. Commonly, variation of the pulse width (PW) is modulated in an FNS system to vary the force generated by the target muscles during a desired movement (Doucet et al., 2012). Model generated muscle excitations (normalized between 0.01 and 1) were mapped to stimulus parameters using saturation ( Sat ) and threshold ( Thresh ) values according to

Stim=Excitation×Sat+Thresh (9)

The values for Sat and Thresh in Equation 9 were previously identified by a physical therapist by the method presented in (Hardin et al., 2007).

Two additional scaling factors were implemented for the optimized pattern during initial stepping trials to account for differences in muscle strength between the model and the study participant. First, the PW for the left-side pattern was too low after scaling by the saturation and threshold values since muscles fatigued prior to finishing a full trial. This was corrected by multiplying PWs for the left side by 1.2. Second, the pattern for muscles on the right-side required a longer period of time to achieve sufficient contraction force, so the right-side time scale was increased by a factor of 50%.

### 2.8 Manually tuned pattern development

The manually tuned pattern was developed by a team comprised of a physical therapist and a biomedical engineer, both of whom were highly experienced and skilled with customizing FNS systems to facilitate walking after SCI. Previously established and published rules for manually setting up and refining temporal patterns of stimulation were followed and formed the basis of their customizing stimulation parameters for the participant (Kobetic and Marsolais, 1994). After preparing an initial pattern based on experience from former studies with the participant and his implanted FNS system (Hardin et al., 2007), the team worked to refine it over multiple sessions on separate days until reaching a plateau in observable symmetry of step length and toe clearance. Because the manually tuned pattern was directly specified as a sequence of PW values, scaling by saturation and threshold was not required for its implementation.

### 2.9 Pattern implementation with participant with SCI

The study participant presented with a C6 level incomplete motor and incomplete sensory (AIS C) spinal cord injury. He had control of his hip extensors and was able to stand with walker support for balance and to control the stance limb during stepping. Having received an implanted neuroprosthesis 20 years prior, the participant was well-practiced in stepping with an FNS system and would use it daily for strength-training exercises. He was not undergoing any physical therapy at the time of this study.

For this study the muscles on the left side were activated via implanted electrodes by the implanted pulse generator. On the right side only the tibialis anterior and vasti were activated by the implanted system, and surface stimulation was applied to additional muscles to augment the implanted muscle set. The short head of biceps femoris and the sartorius were activated by surface stimulation to generate knee and hip flexion. Because the iliopsoas was too deep to access by surface stimulation, the withdrawal reflex was elicited to provide hip flexion; the control signal for this channel was scaled in the same way as the muscle-direct channels.

The participant’s implanted receiver-stimulator system had eight channels (IRS-8) (Smith et al., 1987; Davis et al., 2001) connected to intramuscular electrodes (Memberg et al., 1989). The implanted system received commands and was powered by an external control unit through an inductive coil taped to the skin over the implant site. The external control unit stored the temporal stimulation patterns. All stimulation was controlled by the same external control unit to synchronize implanted and surface stimulation channels. The stimulation waveform was composed of biphasic, constant current, charge-balanced cathodic pulses where the PWs were continuously variable from 0 to 255 microseconds and pulse amplitudes were selectable on a channel-by channel basis. The pulse amplitudes could be set between 0 and 20 mA for implanted stimulation channels and 0 and 100 mA for surface stimulation channels (Mortimer et al., 1980; Smith et al., 1987).

Stimulation frequency was initially set to 16 Hz to minimize fatigue and then doubled after 0.15 s for all muscles except the vasti to ensure sufficient maximum force to complete the desired motions (Doucet et al., 2012). Stimulation frequency returned to 16 Hz on the right side for approximately 30% of the stepping pattern, reducing the potential for additional fatigue (Doucet et al., 2012). On the left side the higher frequency value was maintained for the entirety of the stepping pattern due to the observed lack of stimulated contractile strength of the muscle targets. Frequency modulation was established for the study participant during the development of the manually tuned pattern. We maintained these variations in frequency across both patterns to eliminate it as a variable.

Each step was triggered by an accelerometer-based system for both model-generated and manually tuned stimulus patterns (Shimada et al., 2005; Kotiadis et al., 2010; Foglyano et al., 2016). When the participant pushed the walker forward in preparation for taking a step, an accelerometer mounted on the walker detected motion and activated FNS for the next step.

The participant signed an informed consent form approved by the Institutional Review Board of the Louis Stokes Cleveland VA Medical Center (Reference Number 1591730).

### 2.10 Stepping pattern data collection and analysis

Full body optical motion capture, as described in Section 2.3, and UEE data were collected as the study participant completed overground walking trials. UEE was measured in three dimensions at 1000 Hz sampling frequency with a rolling walker instrumented with loadcells (AMTI, Watertown, MA) in each handle.

Experiments were conducted during a single session with a one-hour rest period between conditions, maintaining surface stimulation electrode placement for consistency. A minimum of five trials were required for each condition. Five trials were completed during the optimized pattern condition, and six trials were collected during the manually tuned pattern condition. A break of at least 10 min was included between trials to allow time for the muscles to recover and minimize the effects of fatigue across both conditions.

A total of 44 left and 45 right steps were collected using the model-optimized pattern, and a total of 55 left and 57 right steps were collected using the manually tuned pattern. Across all collected steps, outliers indicative of failed step detection and toe stubbing were identified and removed based on the interquartile range for medial-lateral walker velocity and toe travel in the anterior/posterior direction. Additional steps were removed from the dataset due to spasm interference and one case of foot crossing. In total 37 left and 39 right steps for the model-optimized pattern and 47 left and 48 right steps for the manually tuned pattern were analyzed.

At the end of each trial, the participant answered questions based on a seven-point Likert-like Usability Rating Scale (Steinfeld and Danford, 1999) and was invited to share additional comments. The usability questions were: (1) Was the task in general difficult, moderate, or easy? (2) Was the task with respect to the upper extremity effort difficult, moderate, or easy? (3) How did the pattern/stepping feel—choppy, smooth, or neither? (4) How stable did you feel during the trial—unstable, stable, or neither? In addition, the physical therapist supporting the trials was asked to rate the amount of assistance required. The scale used for all questions was divided into seven steps from −3 to 3 with negative values associated with poor outcomes. The median, interquartile range, and Wilcoxon rank sum test were computed for each question across trials for each condition.

Gait kinematics and UEE were evaluated during post-processing. Gait data were zero-lag lowpass filtered by a 2^nd^ order Butterworth filter with a 6 Hz cutoff frequency (van den Bogert et al., 2013). Joint kinematics were computed from the marker data in OpenSim via the inverse kinematics tool with a model scaled to the study participant. Across all usable recorded steps, the joint kinematics were stride normalized and ensemble averaged for each pattern. This process was completed on each side due to gait asymmetry. Standard deviation was computed for each trajectory across the normalized time period. Step length, step time, and swing to stance ratio were each averaged across steps per side and the standard deviation was calculated. We computed the UEE resultant force from the three-dimensional force data. The resultant UEE was analyzed by computing the average and standard deviation of the peak values across all usable steps and time-averaged values for each pattern. We analyzed the full gait cycle and the swing phase separately. Statistical comparison was completed using the unequal variance t-test. Normality of the data was confirmed graphically by comparing the data’s empirical cumulative distribution function to the cumulative distribution function for the normal distribution.

## 3 Results

### 3.1 OC problem solution

The *in silico* solution for the last iteration of the OC problem (Predict6) was conducted over a complete two-step cycle. Although only the swing phase portions were extracted for use with the study participant, the full pattern is reported. We define the two-step cycle as being from double stance after right footstrike to double stance after the next right footstrike.

**FIGURE 2:**
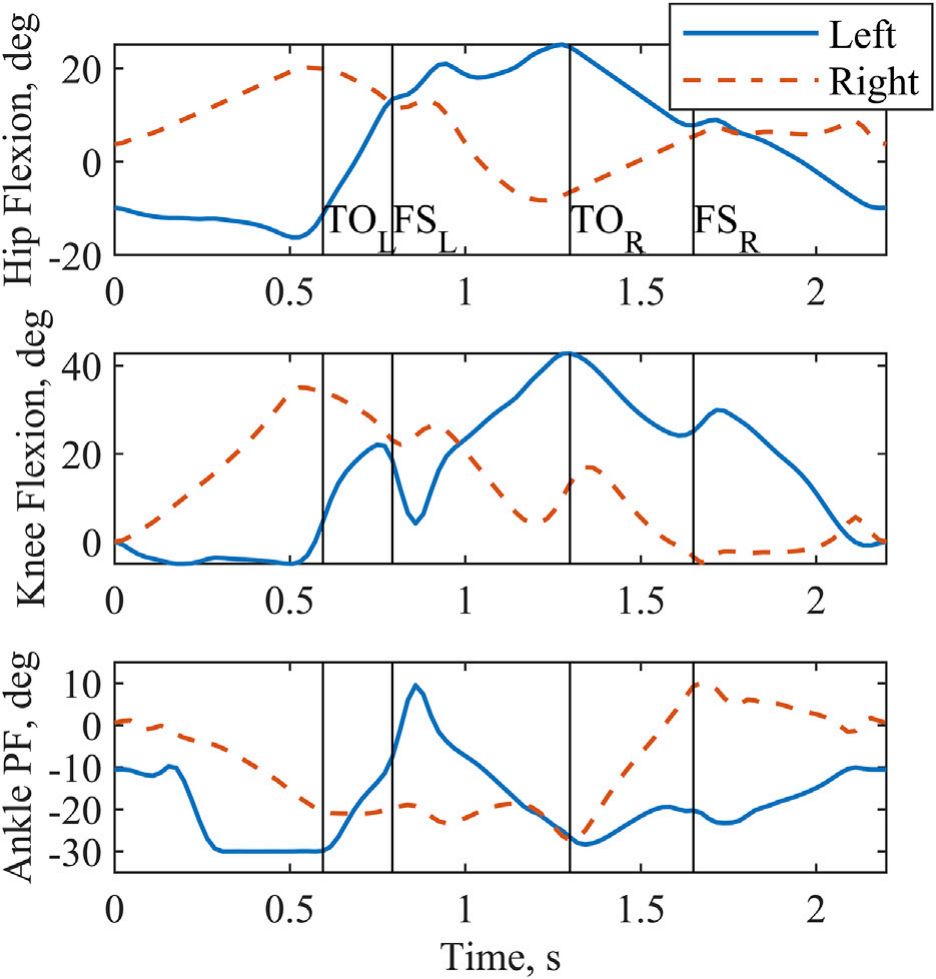
Sagittal plane joint angles output by the optimization for a complete two-step cycle of 2.2 s. Left and right toe off (TO) and footstrike (FS) are indicated by the vertical lines. Plantarflexion is abbreviated as PF.

Sagittal plane hip, knee, and ankle kinematics (Figure 2) from the model-optimized pattern differed from NT gait. Several kinematic features are particularly prominent. During stance phase the supporting hip and knee go into flexion and the supporting ankle dorsiflexes. Additionally, the trunk and arms maintain a slightly posterior posture. During swing, hip hiking without circumduction is indicated. Across the two-step cycle, the optimization meets the symmetry boundary constraints. A video demonstrating the finalized gait with Model P is provided in the Supplementary Material (Supplementary Material S1).

**FIGURE 3:**
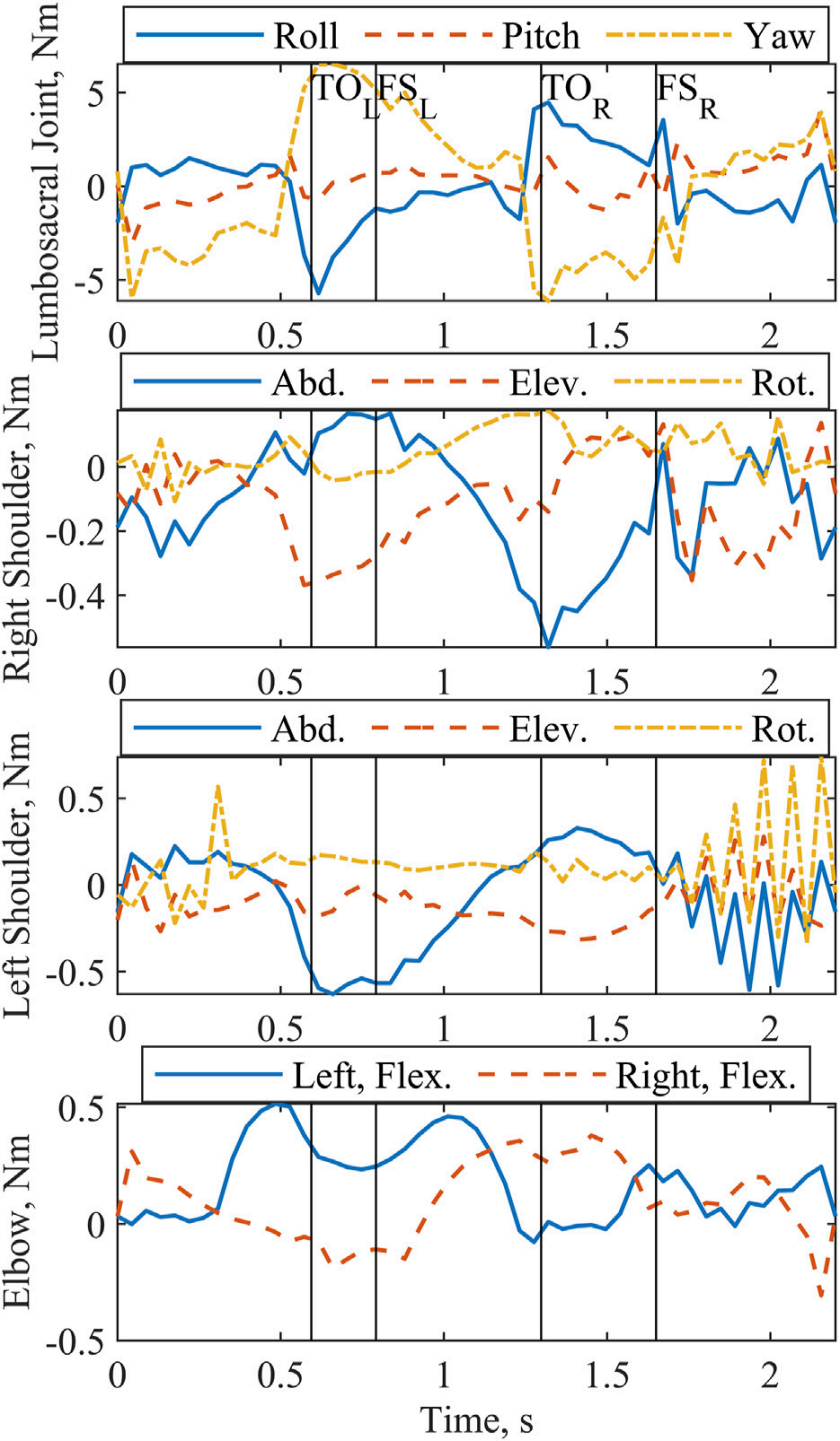
Predicted joint actuator torques for lumbosacral joint, shoulders, and elbows output by the optimization for a complete two-step cycle of 2.2 s. Left and right toe off (TO) and footstrike (FS) are indicated by the vertical lines. Abduction, elevation, rotation, and flexion are abbreviated Abd., Elev., Rot., and Flex.

**FIGURE 4:**
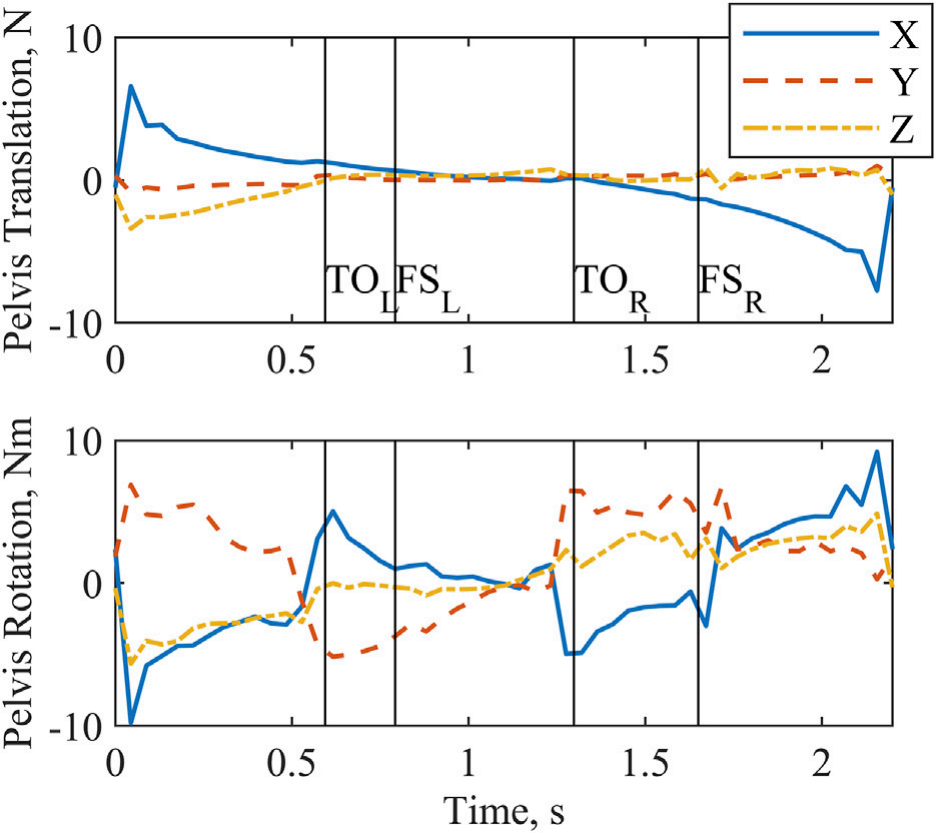
Reserve actuator forces and torques output by the optimization for a complete two-step cycle of 2.2 s. Left and right toe off (TO) and footstrike (FS) are indicated by the vertical lines. X, Y, and Z form a global coordinate system correlating approximately with the anterior, superior, and right-lateral directions, respectively.

All joint actuators (Figure 3) and reserve actuators (Figure 4) were active throughout the two-step cycle. Joint torque actuators at the lumbosacral joint delivered up to 6.5 Nm. The arm joint actuators produced a peak torque of 0.63 Nm. Oscillations near the end of the left shoulder actuator trajectory are likely due to the left shoulder abduction and elevation coordinates approaching the associated speed constraint values. The pelvis translation reserve actuator peak value of − 7.7 N occurs along the X-axis (positive approximately corresponds to the anterior direction), and the pelvis rotation reserve actuator peak value of − 9.8 Nm is about the X-axis.

**FIGURE 5:**
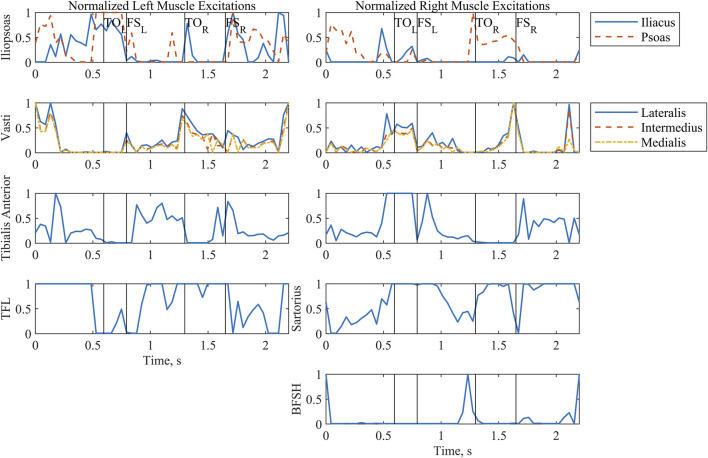
Normalized muscle excitations output by the optimization for a complete two-step cycle of 2.2 s. Where the model was symmetrical, muscle groups have been arranged by row. The first column includes all left-side muscles, and the second column includes all right-side muscles. Left and right toe off (TO) and footstrike (FS) are indicated by the vertical lines. Tensor fasciae latae is abbreviated as TFL. Biceps femoris short head is abbreviated as BFSH.

Patterns selected by the optimization for the muscle excitations (Figure 5) emphasized transient spikes. Notably, the hip flexors (iliopsoas, tensor fasciae latae, and sartorius) were particularly active during stance. The vasti of the supporting limb are active during swing phase, as expected. Furthermore, the vasti are active on the swing limb side during the latter part of swing phase to extend the knee in preparation for footstrike. Hip flexor activity peaks during swing. In addition, the excitation profiles for the iliopsoas and vasti muscle groups evidence the effects of the muscle synergy cost function subterm; the control signals for the individual muscles in the groups follow a similar trajectory throughout much of the two-step cycle.

### 3.2 Implemented patterns

**FIGURE 6:**
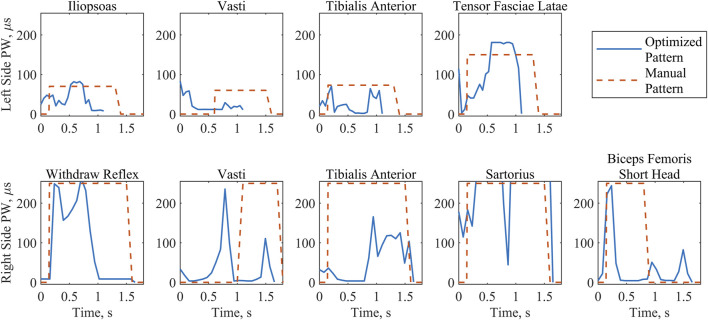
Stimulation PW values implemented for the optimized and the manually tuned patterns during swing phase. Where the electrodes were symmetrical, muscle groups have been arranged by column. The first row includes all left-side muscle patterns during the left step. The second row includes all right-side muscle patterns during the right step. Note the differing durations of swing between patterns and left and right sides, with the optimized pattern consistently shorter than the manual pattern.

The raw PW values for the model-optimized and manually tuned patterns illustrated the distinct differences between the patterns (Figure 6). While peak values for both patterns were similar due to the previously described scaling process, PW values (Figure 6) throughout the profiles diverge, causing notable differences in the total charge delivered (Table 4). With the exception of the right sartorius, the total charge delivered with the optimized pattern of PW values for each muscle was smaller than that of the manually tuned pattern. This difference ranged from a 35% reduction for the left tensor fasciae latae to an 80% reduction for the right tibialis anterior. For the right sartorius the optimized pattern charge delivered was 39% greater than the manually tuned pattern.

**TABLE 4:** Charge delivered per swing phase in μC .

Muscle Group	Optimized pattern		Manual pattern	
	Left	Right	Left	Right
Iliopsoas/Withdraw Reflex	28.1	294.7	56.0	585.0
Vasti	9.5	21.1	19.2	55.0
Tibialis Anterior	17.5	38.9	58.4	195.0
Tensor Fasciae Latae	77.8	-	120.0	-
Sartorius	-	948.7	-	682.5
Biceps Femoris Short Head	-	94.5	-	300.0

### 3.3 *In vivo* stepping performance

The number of analyzable steps per trial for each condition is reported (Table 5) to evaluate the effects of fatigue on trial length. The optimized pattern condition was tested during the first part of the single-day session. Due to spasms, the fifth trial was cut short.

**TABLE 5:** Number of steps analyzed for each condition. Note that the optimized pattern was tested first.

Trial #	Optimized pattern	Manual pattern
Trial 1	22	21
Trial 2	15	22
Trial 3	17	10
Trial 4	19	13
Trial 5	3	10
Trial 6	-	19

**FIGURE 7:**
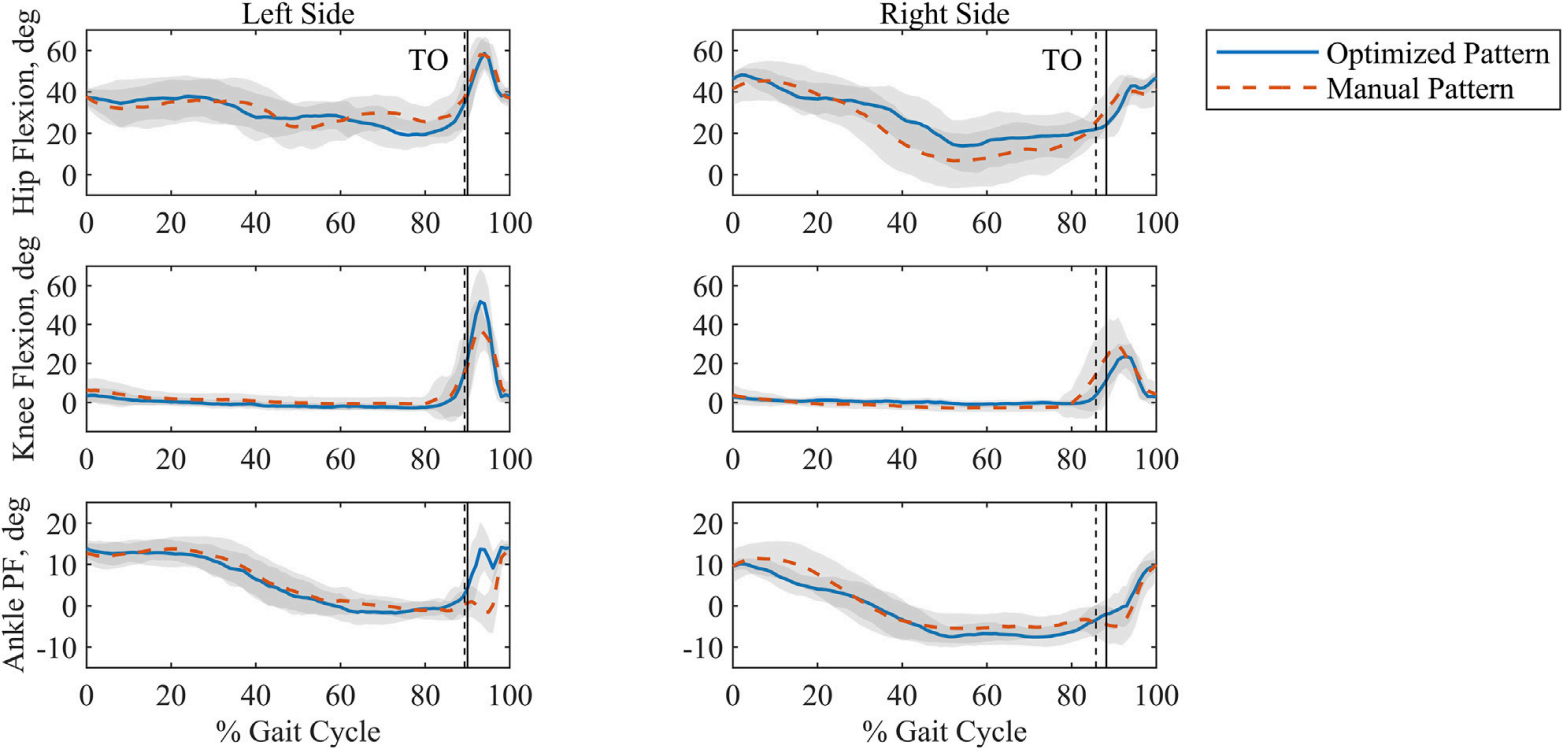
Ensemble averaged, time-normalized sagittal plane joint angles for the optimized and manually tuned stimulation patterns for the left and right sides with standard deviation shaded in grey. Percent Gait Cycle is represented as being from footstrike to footstrike per side. Toe off (TO) is indicated by the vertical lines. The solid vertical line is associated with the optimized pattern, and the dashed vertical line is associated with the manual pattern. Plantarflexion is abbreviated as PF.

Joint kinematics measured experimentally were compared between stimulation patterns (Figure 7). Both patterns produced similar hip flexion and ankle plantarflexion profiles. Across all joint angles, the left side showed larger joint angle excursions than the right side under both stimulation patterns. In addition, a posture of hip flexion was maintained for both patterns and sides throughout the gait cycle. The highest value for knee flexion angle (51.7 ° ) occured on the left side for the model-optimized pattern.

The values measured for step time and length are similar for both patterns (Table 6). Because they were approximately symmetrical within a given pattern, we can estimate gait speed (Singleton et al., 1992); it was 0.032 m/s and 0.031 m/s for the optimized and manually tuned patterns, respectively. Comparing swing and stance time shows that right swing is consistently longer than left swing for both patterns. Additionally, the optimized pattern resulted in a statistically significant decrease (p < 0.005) relative to the manual pattern for right swing time (Table 6).

**TABLE 6:** Comparison of step time, ratio of swing to stance time, and step length. One standard deviation is shown in parentheses. T-test indicated no statistically significant differences between either pattern or side for step time and step length. Statistically significant differences in swing to stance ratio are paired by matching symbols.

Measure	Optimized pattern		Manual pattern	
	Left	Right	Left	Right
Step Time, s	16.85 (3.04)	16.80 (3.18)	18.09 (4.22)	17.82 (4.52)
Swing to Stance Ratio	0.13 (0.03)^†^	0.15 (0.04)^†‡^	0.14 (0.04)*	0.18 (0.05)*^‡^
Step Length, m	0.539 (0.062)	0.538 (0.059)	0.556 (0.052)	0.564 (0.064)

* p < 0.001, ^†^ and ^‡^ p < 0.005.

**FIGURE 8:**
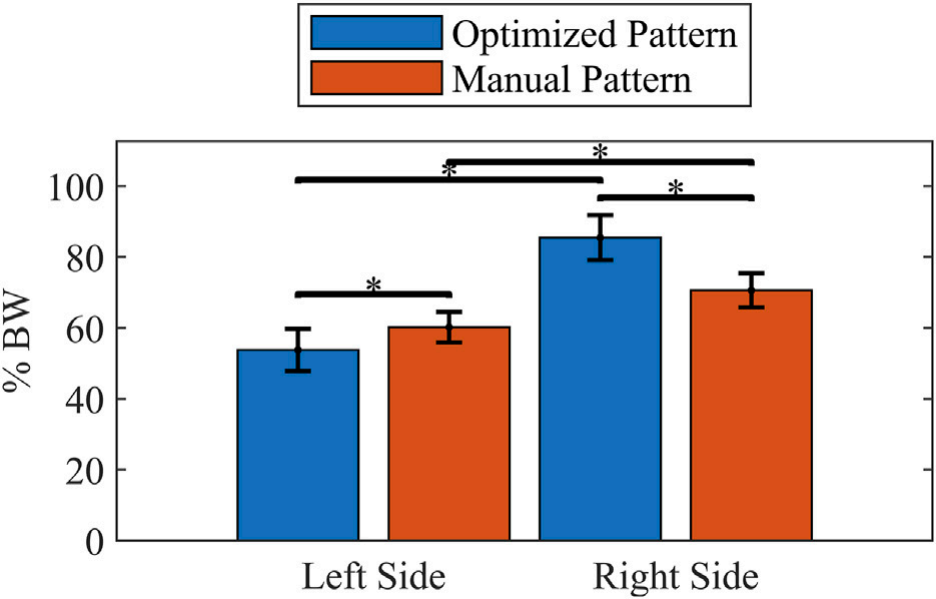
Comparison of mean peak UEE during swing phase for each pattern and side. Forces scaled to percent body weight (% BW). Error bars show one standard deviation. * indicates p < 0.001.

The manually tuned pattern resulted in a lower peak UEE (p < 0.001) than the optimized pattern (Table 7). However, the average force across the gait cycle was comparable between patterns. When isolating the UEE required during swing for each side, it was noted that the left peak UEE for the optimized pattern was less (p < 0.001) than for the manually tuned pattern (Figure 8). The optimized right-side pattern UEE was higher (p < 0.001) than the manually tuned right-side pattern UEE (Figure 8). Additionally, for both patterns the right UEE was higher (p < 0.001) than the left UEE (Figure 8).

**TABLE 7:** Peak and average UEE per gait cycle. One standard deviation is shown in parentheses. Statistical comparison conducted across patterns.

UEE Measure	Optimized pattern	Manual pattern
Peak UEE, N	444 (32)	368 (23)*
Mean UEE, N	192 (16)	186 (17)

*p < 0.001.

### 3.4 Study participant feedback

**FIGURE 9:**
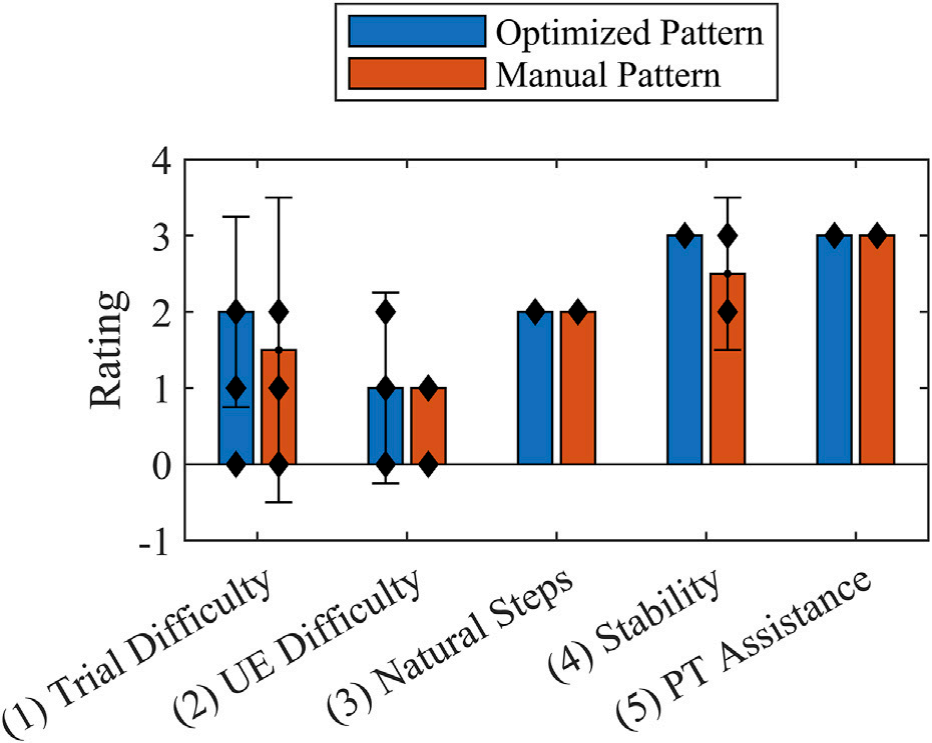
Usability Rating Scale median values reported by the participant (Questions 1-4) and physical therapist (Question 5) at the end of each trial for the optimized stimulation pattern and manually tuned stimulation pattern. Error bars show interquartile range. The individual data points are indicated by black diamonds. The Wilcoxon rank sum test indicated no statistical significance between patterns.

The participant similarly rated the optimized pattern and the manually tuned pattern (Figure 9). The physical therapist rated both patterns equally as requiring the lowest level of assistance. Additionally, the study participant indicated a strong association between the PW specified for the left side by the optimized pattern and increased comfort, stating that “the step felt natural”. He further explained that he only needed to consciously initiate the step and the rest of the action would follow. This was in contrast to the right step for the optimized pattern, during which he reported feeling the progression of the FNS system activating each muscle individually. In the latter case the participant felt that he could coordinate well with the pattern because he could distinctly feel each muscle being activated, which he indicated was similar for the manually tuned pattern. The participant offered that he would choose to combine the natural-feeling optimized pattern on the left side with the manually tuned pattern on the right side.

## 4 Discussion

In this study we developed an *in silico* FNS stepping pattern that included only the muscles used for FNS for a specific individual with an incomplete SCI. The OC approach was implemented with as few cost function terms and constraints as possible to identify a solution that minimized the stimulation PW and UEE. The results were compared to a standard, manually tuned stimulation pattern, indicating the potential for reducing UEE and muscle effort while improving participant comfort while stepping.

### 4.1 OC problem solution

In setting up the predictive OC problems, we allowed atypical kinematics by using an NT-gait based initial guess but no tracking component. This approach was selected because of the expectation that the combination of a reduced muscle set and minimizing muscle excitations could result in a unique gait pattern. The kinematics generated by the optimization emphasized an anterior flexing strategy for generating forward propulsion, which is consistent with bipedal gait (Wang and Srinivasan, 2014), though this is an extreme representation. Because propulsion was anticipated to be generated primarily by the participant’s interaction with the walker due to the lack of activation of the plantarflexor muscles (Awad et al., 2020) and because the swing phase alone would be extracted for analysis, the anterior flexing propulsion strategy was not penalized. Along with the propulsion approach, the optimizer elected a posterior posture of the trunk and arms, providing a counterbalance in the anterior/posterior direction. The third notable gait characteristic was hip hiking without circumduction that helped to provide clearance for the foot. This feature is consistent with the absence of major hip abductor muscles (Table 1).

By selecting this gait strategy for the reduced muscle model, the optimizer minimized the effort required from the arm and lumbosacral joints, producing physiologically reasonable values. NT shoulder moments during walking have been reported between 2.2 Nm and 12 Nm (Meyns et al., 2013). Our peak value of 0.63 Nm indicates that the optimization found a comparatively low torque solution. Even though the values were low, we note that the solver did elect to use the arm actuators instead of producing a fully passive solution; this feature is consistent with the consensus that arm motion is not entirely passive (Meyns et al., 2013). For all three lumbosacral DOF, Samadi et al. recorded peak torques during NT gait of less than 0.1 Nm/kg between the L2 and L3 vertebrae (Samadi et al., 2020), which are similar to the values reported. This solution overall suggests the potential for shifting effort expenditure from the upper extremities to stimulation induced contractions of the paralyzed lower extremity musculature, even with a reduced muscle set.

Considering that the physical parallel to the reserve actuators is participant interaction with the walker, analysis of these actuators’ magnitude and timing also supported the conclusion that the OC solution maximized the effort expenditure of the muscles over the use of reserve actuators. The peak force applied by the reserves to the pelvis was 1.0% of body weight, confirming that body weight was essentially fully supported through the legs. The timing of the reserve actuators suggested that their primary use was twofold. First, propulsion was indicated by activity of the torque actuators occurring just prior to the model taking a step. Second, posture control was evidenced because at the beginning and end of the two-step cycle the peak action of the force and torque actuators are associated with initiating and arresting motion, respectively, to enforce the zero joint velocity state constraint $C_{rest}$ . Therefore, the reserve actuator activity was appropriately small, indicating that it was effective in supporting the solution of the optimization problem and representing the study participant’s use of a walker without injecting excess energy and skewing the results.

Muscle excitations were effectively minimized and provided the majority of the effort required to achieve the selected gait strategy. Transient bursts of muscle activity appear to be the primary means of reducing overall muscle effort. The activation dynamics accounted for in the model serve as low pass filters, resulting in the muscles generating an overall smoother force output than the profiles identified for the muscle excitations. Remarkably, the solution included substantial hip flexor excitations for the stance limb. This activity, however, is consistent with the kinematic strategy as it reinforces a motion that would augment the propulsive forward flexion. Furthermore, due to the limited lumbosacral pitch joint actuator torque, we can conclude that the actions of the psoas portion of the iliopsoas on the lumbar spine also likely support the trunk posteriorly and stiffen the vertebrae to counterbalance the forward motion. When evaluating the muscle excitations selected for the iliopsoas, the effects of the muscle synergy cost function subterm are mixed; the previously mentioned tasks, forward propulsion and balancing the trunk, prevailed over the muscle synergy cost function during much of the two-step cycle. However, the influence of the muscle synergy cost function subterm on the muscle excitations is clearly illustrated for the vasti, which act together to extend the knee.

Reduction of the PW values has been associated with increased comfort (Doucet et al., 2012). When comparing the stimulation patterns used during stepping trials, the optimized pattern resulted in a reduction of the charge delivered for all muscles except the right sartorius, which might reflect suboptimal synergy constraints for that muscle. Similar peak PW values between patterns indicate that the reduction of charge delivered for the optimized pattern is due to varying the PW values throughout the step, in contrast to the manually tuned pattern in which they remain high during the full period of a given muscle’s activity. While the same tactic could be implemented manually, the number of combinations to try could be overwhelmingly large, suggesting the benefit of an *in silico* approach.

### 4.2 Experimental performance

Due to the irregularity of stepping under FNS, standard measures of fatigue based on variability could not be evaluated. However, Table 5 provides evidence that fatigue did not become a major influence in the experimental results. The length of the first trial of the day (Optimized Pattern Condition, Trial 1) is comparable to the length of the last trial of the day (Manually Tuned Pattern, Trial 6). Furthermore, short trials appear at random intervals.

Comparison of joint kinematics between the two patterns suggests a consistent strategy during stance and limited differences under stimulation-driven motion. Because stance phase was controlled volitionally by the study participant, it follows that similar features would be reflected for both patterns. In general, swing joint angles are greater for the left side than the right side, regardless of the stimulation pattern. This might indicate a difference in the stimulation methods (surface vs implanted). Hip flexion was also noted throughout the gait cycle. In stepping, forward-leaning flexion is characteristic of walker use for individuals with SCI due to the need to support bodyweight in addition to maintaining balance (Melis et al., 1999). Lastly, we observe that the knee angle generated by the optimized pattern for the left side nears NT peak values (Winter 1984).

Evaluation of step time and length for the optimized and manually tuned patterns further confirms the similar kinematic features for both patterns. No statistical significance could be assigned to these measures, and the values for step time and step length within a given pattern are quite close, suggesting a degree of gait symmetry for these measures (Singleton et al., 1992). When evaluating the ratio of swing to stance time, the right swing was longer than left swing across patterns, which could be due to a faster muscle response time for the fully implanted electrode architecture used on the left side over the mixed architecture of the right side. In both patterns dwell time in double stance has a clear effect on walking speed. Even though a statistically significant decrease in right swing to stance time ratio was seen when comparing the optimized pattern to the manual pattern, the change in right step time was not statistically significant. Considering these results together indicates the effect of the participant’s gait style that included pausing during double stance to reduce spasms. The similarities in joint kinematics, step time, and step length across patterns indicate that PW can be effectively decreased by an *in silico* solution while maintaining gait quality relative to a manually tuned pattern.

Measures of UEE demonstrate the potential for developing patterns *in silico* that reduce the UEE required for walking with FNS. While the peak UEE for the manually tuned pattern was less than the optimized pattern, the average UEE was similar, indicating that peak force might be an important but insufficient measure of the effort required by the participant. It is also of interest to compare left and right sides across patterns. This comparison accounted for only the effort required during a step (swing phase) to better isolate the effects of a given tuning method. The left peak UEE measuring less than the right peak UEE during swing indicates the potential for a pattern developed *in silico* by OC to reduce peak UEE, even though the current optimized pattern did not achieve an overall reduction. The swing-only comparison also made it evident that for both patterns right UEE was greater than left UEE. One might conjecture that the use of combined stimulation methods on the right side (as opposed to implant only on the left side) is the source of this increase. However, because the right-side UEE associated with the optimized pattern is higher than the right-side UEE associated with the manually tuned pattern, we reject this reasoning and recommend further experiments to verify whether stimulation method is the source of the higher right over left UEE observed across patterns. Though a direct comparison cannot be made due to the differing locations of force application between the model and experimental cases, we note that when comparing the peak force values of the predicted OC reserve actuators and the measured UEE the participant exerted upwards of 50 times more force, which we primarily attribute to the less erect posture of the subject during the experimental condition.

Feedback comparing and contrasting both stimulation patterns provided by the participant indicated either no difference between patterns or a slight preference for the optimized pattern. Questions targeting effort in general, UEE in particular, and stability recorded on a Likert-like showed no statistical difference, demonstrating the capacity of an OC-based solution to match a manually tuned pattern. The rating for general effort is consistent with the optimized pattern’s overall reduction in muscle activation by FNS. Additionally, the UEE rating suggested that the differences in UEE peak forces between patterns could have been undetectable by the participant. The stability of both patterns was validated by the physical therapist; the rating for all trials indicated that the level of assistance needed was contact guard assist, whereby constant contact with the gait belt was only a preventative safety measure. When asked open-ended questions about the pattern, the participant confirmed the value of decreased PW levels (Figure 6) by stating that the left step of the optimized pattern felt natural.

### 4.3 OC in clinical practice

A participant-informed model was employed for the final iteration of the OC problem. We assumed that the model must include the muscles specific to the individual using the FNS system, but no further participant-specific refinement was done. This approach resulted in a workable pattern that did not require information from preliminary laboratory sessions such as contractile properties, muscle strength, body geometry, and stimulation reaction time measurements. Only two walking trials at the beginning of the session were necessary for scaling the model-optimized stimulation pattern. While the pattern might be improved by further model personalization, the sufficiency of this degree of personalization opens the opportunity of holding fewer, less intensive laboratory sessions.

Furthermore, for clinical implementation we suggest that the work done with Model G might not require repeating. For future study participants the addition/removal of muscles for developing Model P can be programmed into a graphical user interface (GUI) that also includes a switch indicating whether the muscle set is symmetrical or not, which would subsequently determine whether a single stride or two-step gait cycle needs to be optimized. After running the optimization prior to clinical time with the patient, it would be sufficient to tune the strength of the stimulation.

As gains in FNS technology continue, the number of stimulus channels (and muscles able to be selectively recruited) will increase. This progress raises the question of how to efficiently coordinate more muscles as the parameter space expands. Unlike single-joint studies, our 3D model captures multi-muscle coordination. Shifting the pattern generation process to an *in silico* approach could help to address this dilemma as optimal control problems for musculoskeletal models containing more muscles are readily solved. For example, Dembia et al. present an OpenSim model with 80 lower-limb muscles (Dembia et al., 2020).

### 4.4 Future considerations

There are several limitations to the current study. Clearly, a single participant is insufficient to generalize the *in silico* approach presented here for the larger population of individuals with incomplete SCI, which is by nature heterogeneous. Future work should include a broader pool of participants with various levels of injury, extent of paralysis, and available muscle targets for activation, allowing for more robust statistical comparison of each tuning condition. All participants capable of stepping with FNS could be considered under our framework, although future studies might need to separate participants into groups according to injury level. The data provided by the current study will inform a power analysis in planning for a larger study.

Muscle spasms with clonus were particularly prevalent during some trials and seemed to increase later in the day during the second session. Efforts were made to remove steps from the dataset where stimulation did not overcome this involuntary muscle activity and interfered with step progression. With the current study it is unclear whether this increased activity was related to the stimulation pattern being tested, changes to joint angle and sensory inputs to the spinal circuitry below the level of injury, fatigue, or other factors. Also, it can be noted that the left tensor fasciae latae stimulation profile from the optimized pattern exhibited a difference in timing but not pulse width values when compared to the manually tuned pattern. While this is not an exact representation of the OC problem output, we anticipate that it has a minimal effect on the outcome, though it might be a consideration for future work.

This study focused on reducing stimulation levels through modulating the PW. NT muscle isometric force values were used in the model, and the scaling of the predicted excitation pattern to account for reduced muscle strength after SCI, which is typically less than 50% of NT values, was completed during the experiment session. This potential limitation will be assessed in future work by exploring experimental techniques such as using dynamometers like the Biodex System 4 to quantify the subject- and muscle-specific contractile properties with FNS to achieve a better understanding of whether it is necessary to estimate muscle strength at that level of detail for each participant.

Upon completing this study, two directions for extending it are of particular interest. First, development of an optimization goal or other conditions that eliminate the forward leaning propulsion strategy should be pursued. To extend this work to individuals with complete SCI or those with incomplete SCI and less hip extensor control requires eliminating this propulsion strategy because stability during stance is a priority to this population. The solution to this issue might include adding a model of the walker assistive device to the simulation which would direct the solver toward solutions consistent with a more reasonable trunk pose. Second, while PW modulation effectively improves comfort, minimizing fatigue might be a higher priority and more important consequence of the optimal control approach. Future investigations could explore methods of modeling stimulation frequency in addition to PW modulation and cost function terms that target fatigue (Doucet et al., 2012).

After refining the optimal control problem and its open loop implementation, we will integrate the results into a closed loop FNS system. The open loop muscle activation pattern determined by solution of the optimal control problem has an associated CoM trajectory. A feedback controller can be used to compute differential activations to be added to the baseline (feedforward) muscle activations should deviation from that CoM trajectory occur due to external or internal (fatigue) perturbations. While a similar approach could be taken with a manually specified muscle activation pattern, this often results in over specification of the stimulation levels required to achieve a step. By using the optimal control solution, we will maintain the benefits of decreased stimulation levels while including a feedback component.

## Data Availability

The raw data supporting the conclusions of this article will be made available by the authors, without undue reservation.

## Supplementary material

SUPPLEMENTARY VIDEO S1 Video illustrating the gait produced by solution of the optimal control problem in OpenSim.
